# Massive Pulmonary Hemorrhage from Bronchial Varix

**DOI:** 10.1155/2020/9175785

**Published:** 2020-04-04

**Authors:** Michael Agustin, Scott Shay, Jose Gonzalez, Pei Liu, Nancy Lentz, Anna Shapiro, Nat Dumrongmongcolgul, Michael Torres, Vasin Jungtrakoolchai

**Affiliations:** Guam Regional Medical City Intensive Care Unit, 133 Route 3, 96929 Dededo, Guam, USA

## Abstract

Bronchial varix is a rare pulmonary disorder which may lead to life-threatening hemorrhage. Diagnosis is difficult because of the subtle abnormalities on radiographic and bronchoscopic examination. We present a case of massive hemoptysis from a bleeding bronchial varix. In the absence of immediate complex endobronchial therapy in the island of Guam, this case was initially managed with nebulized and intravenous tranexamic acid. This was followed by endobronchial blockade of the bleeding airway with endobronchial epinephrine instillation. Selective bronchial artery embolization alleviated the acute-phase bleeding. Prone positioning was initiated due to severe hypoxia after blood clots compromised the patency of bilateral bronchial airways. Prone ventilation was employed for 17 hours for 2 consecutive days with intermittent bronchoscopic forceps extraction of airway blood clots while in the prone position. These maneuvers resulted to improved lung ventilation and oxygenation. The patient underwent bronchial sleeve resection surgery for definitive management.

## 1. Introduction

Pulmonary varix is a rare pulmonary venous disorder characterized as aneurysmatic venous dilatation. There are very limited cases reported in the literature. Pulmonary varices can be congenital or acquired and isolated or associated with varices in other organs [1]. Congenital varices may coexist with other congenital heart diseases. Diseases with increased pulmonary vein pressure such as mitral valve disease or distal occlusion of the pulmonary veins, liver cirrhosis, or emphysema are associated with acquired forms of pulmonary varices [1]. The main complications of pulmonary varices are rupture, hemoptysis, and thrombosis with systemic embolism.

We present a case of a massive hemoptysis from a bleeding left bronchial varix. In the absence of a complex pulmonary interventional therapy at our facility in the island of Guam, tranexamic acid nebulization and selective bronchial artery embolization were initiated. These procedures were followed by multiple manual forceps extraction of large airway blood clots via bronchoscopy while the patient is on prone position ventilation. Selective bronchial artery embolization may have decreased the flow of the feeding vessel leading to decreased venous congestion. The effect of prone positioning on the natural airway drainage may have led to the improvement of posterior lung ventilation. Embolization of a bronchial artery on a bleeding bronchial varix has been reported by Shweihat and Zoby [2]. Likewise, this hypothesis of enhance airway drainage with the prone position in pulmonary hemorrhage is discussed only on very limited case reports [3, 4].

## 2. Case Presentation

**Figure 1:**
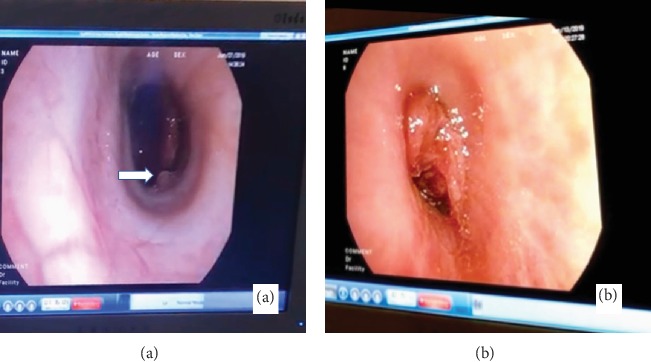
(a) Bronchoscopic findings showing polyp-like lesion on the left main bronchus about 2 cm from the carina. (b) Blood clots proximal to the polyp-like lesion suggestive of active bleeding site.

**Figure 2:**
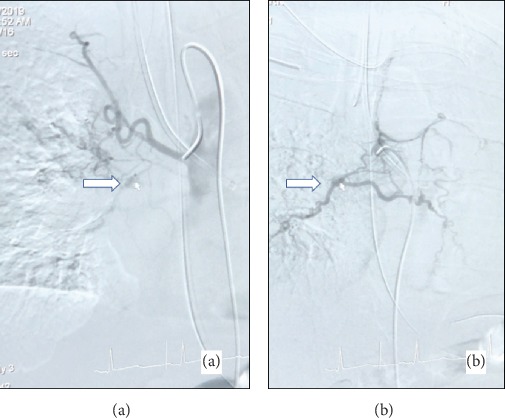
(a) Selective right bronchial artery angiography showed collateral flow from the right bronchial artery to the left bronchial artery supplying the presumed bronchial polyp or abnormal blood vessel (white arrow). (b) Selective left bronchial artery angiography showing left arterial supply to the lesion with collateral flow from right bronchial artery feeding presumed polyp-like lesion or abnormal vessel (white arrow).

**Figure 3:**
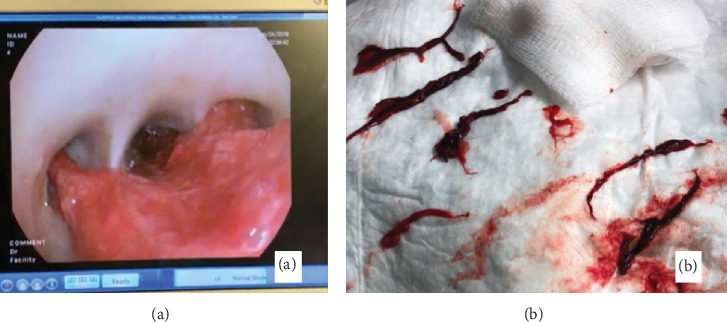
(a) Bronchial airway obstruction from blood clots. (b) Manually retrieved blood clots from large airway via forceps extraction.

**Figure 4:**
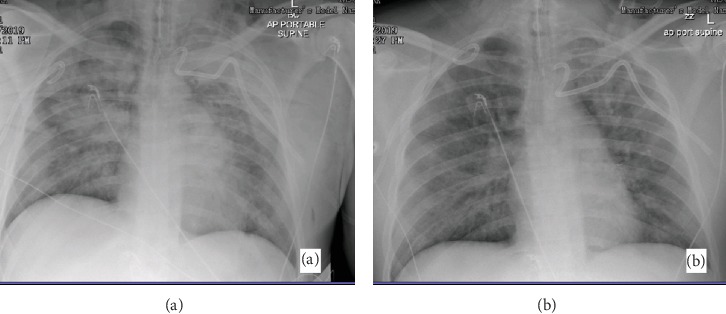
(a) Chest X-ray preprone positioning showing bilateral air space disease with patchy bilateral consolidation from massive spillage of blood from bleeding left bronchial varix and atelectasis. Bilateral pneumothoraxes with placement of bilateral chest tubes. (b) Forty-eight (48) hours post prone positioning with mechanical clot extraction showed interval improvement of airspace disease.

A thirty-eight-year-old male with a history of vape use was admitted for massive hemoptysis of about 500 ml on two episodes. Nebulized and intravenous tranexamic acid was given. Bedside bronchoscopy localized the lesion on the left lung with polyp-like lesion versus an abnormal vessel on the left main bronchus about 2 cm from the carina (Figure 1(a)). Blood clots were also noted proximal to the polyp-like lesion suggestive of the primary site of active bleeding (Figure 1(b)). Endobronchial blocker was placed on the left main bronchus followed by the instillation of endobronchial epinephrine. Bronchial artery angiography showed collateral flow from the right bronchial artery to the left bronchial artery supplying the presumed bronchial polyp or abnormal blood vessel (Figures 2(a) and 2(b)). Gelfoam slurry embolization was performed on the right bronchial artery with follow-up angiography demonstrating occlusion of the collateral vessels from the right bronchial artery. Procedures on the left bronchial artery were aborted due to acute upward angulation of the vessel and the risk of embolizing the anterior spinal artery. Following the successful right bronchial collateral artery embolization, endobronchial bleeding stopped, and the patient was successfully extubated. A week after, repeat chest computed tomography (CT) revealed bilateral pulmonary embolism (PE). The patient had another episode of massive hemoptysis with almost one liter of expectorated blood. Left bronchial artery embolization with Gelfoam slurry was performed via left brachial artery approach. Multiple bleeding ensued which required fourteen (14) bronchoscopic clearings of airway blood clots via forceps extractions (Figures 3(a) and 3(b)). Pulmonary consolidations on bilateral lungs also developed which were considered to be pulmonary infarctions and/or atelectasis from plugging of airways with blood clots (Figure 4(a)). The patient's persistent severe hypoxia led to cardiac compromise, and the patient went to pulse electrical activity arrest. Increased positive expiratory pressure (PEEP) in the ventilator also led bilateral pneumothorax requiring bilateral chest tube placement (Figure 4(a)). Given the severe hypoxia, the team decided for prone positioning with paralytics. Prone ventilation was done for seventeen (17) hours for two consecutive days with intermittent prone bronchoscopies for manual forceps extraction and therapeutic aspiration of bronchial blood clots in large airways. The patient's oxygenation improved 48 hours post prone positioning with an interval decrease in airspace opacities on repeat chest X-ray (Figure 4(b)). The patient was extubated after five (5) days. The patient was medically evacuated to a tertiary university center in California where he underwent successful left bronchial sleeve resection surgery. A pathology report on the bronchus showed dilated arteries with mural thrombus in the abnormal artery consistent with bronchial varix.

## 3. Discussion

Pulmonary varix is a rare pulmonary venous disorder with fewer than 100 cases reported in the literature. Diseases associated with increase pulmonary venous pressure may lead to the aneurysmatic dilatation of one or more pulmonary veins. Congenital varices develop during the embryonic period and may coexist with other congenital heart diseases. Acquired pulmonary varices can develop not only due to portal hypertension but also due to pulmonary venous obstruction. Cases of both mitral stenosis and pulmonary vein stenosis have led to the occurrence of pulmonary varices [1, 5]. Other conditions associated with dilatation of the pulmonary veins such as pulmonary arteriovenous fistulas and hepatopulmonary and scimitar syndromes must be ruled out.

Varices are very rare in the airways. In our case, the diagnosis was challenging as there were no definite radiographic findings and very subtle abnormalities on bronchoscopic evaluation. The patient's CT findings showed normal-appearing pulmonary arteries. The angiographic finding showed possible aberrant vessel feeding the lesion on the distal left main bronchus and was not typical of an arteriovenous malformation. The criteria for the angiographic diagnosis for pulmonary varices were established by Berecova et al. and Batram and Strickland [6, 7]. There should be normal pulmonary arteries, absence of pulmonary arteriovenous fistulae, simultaneous filling of varicose and normal veins, varices draining into the left atrium, and prolonged emptying compared to normal veins, and the dilated and tortuous varices are central and near the hilum with normal peripheral veins [1, 6, 7]. In a review of 71 published cases, pulmonary varices can be of three types, namely, saccular, tortuous, or confluent type [1]. In one case report, the use of Narrow Band Imaging (NBI) can facilitate the recognition of abnormal superficial and deep mucosal or submucosal vessels due to the different penetration of the used emitted wave lengths [2].

Treatment is usually unnecessary unless varix rapidly increases in size or complication such as hemoptysis, thromboembolic disease, or rupture occurs. In the report by Shweihat and Zoby on a young patient with recurrent hemoptysis, embolization of the artery resulted in the control of the hemoptysis and reduction in size of the varices on subsequent bronchoscopic evaluation [2]. Arterial embolization indicates that the increase in flow from the feeding vessel is the predominant pathology on a patient's bronchial varices. Bronchial artery embolization would possibly lead to a subsequent decrease of bronchial venous congestion. In our case, we have seen remarkable reduction of bleeding after selective bronchial artery embolization. In a cirrhotic patient who develops bronchial varices, sclerotherapy has been considered for acute hemorrhage [8]. Overall, no well-validated therapy specific to bronchial varices has been developed.

Severe hypoxia may also occur with severe pulmonary hemorrhage. In our case, the patient's massive bleeding for bronchial varix caused massive spillage of blood all over the airway causing worsening V/Q mismatch. The appearance of bilateral consolidations may be due to pulmonary infarctions or atelectasis from blood clots plugging the airways. There are very limited case reports of acute large vessel pulmonary hemorrhage managed by placing the patient in the prone position [3, 4]. These reports emphasized the advantage of the prone position in removing airway secretions specially the blood. Bleeding from the main bronchus may spread across the entire airway causing hypoxia with and without hypoventilation. Blood clots will result to a physical barrier in the large and small airways as well as a barrier to gas diffusion in the alveolar level. Blood drains posteriorly blocking dependent airway in the supine position which will result to worsening V/Q mismatch on the dorsal units. The effect of gravity plays an important role on the propensity of the posterior part of the lung to be affected mainly by pulmonary hemorrhage. The prone position will encourage the flow of blood that mimics the natural drainage position of the superior segments of the lower lobe [4, 9]. This position provides better clearing of airways that helps improve ventilation and lung volumes. Clearing the airways to these lobes will allow greater ventilation and higher tidal volumes leading to overall improvement of oxygenation. In our case, upon turning the patient into the prone position, gas exchange improved dramatically and immediately. We have mechanically aided large airway clearance by doing intermittent therapeutic aspiration with bronchoscopy while the patient is in the prone position.

In conclusion, bronchial varix is a very rare pathology which may lead to a massive hemorrhage. In the absence of immediate complex pulmonary interventional therapy in our island, this case was managed emergently with nebulized and intravenous tranexamic acid. This was followed by endobronchial blockade and endobronchial epinephrine instillation. The patient's acute bleeding episodes responded well with bronchial artery embolization indicating that the increase in flow is the possible predominant pathology of this bronchial varix. Prone positioning provided improved oxygenation from improved airway clearance of blood clots and possible recruitment of posterior basal lungs. We suggest further studies on the benefit of bronchial artery embolization in cases of bronchial varices. In addition, it may be appropriate to explore other utilities of prone positioning with mechanical clot extraction in severe hypoxia secondary to pulmonary hemorrhage. Definitive treatment with bronchial sleeve resection surgery was done in this case to prevent recurrent bleeding.
